# Social Media for ImpLementing Evidence (SMILE): Conceptual Framework

**DOI:** 10.2196/29891

**Published:** 2022-03-09

**Authors:** Junqiang Zhao, Gillian Harvey, Amanda Vandyk, Wendy Gifford

**Affiliations:** 1 School of Nursing Faculty of Health Sciences University of Ottawa Ottawa, ON Canada; 2 College of Nursing and Health Sciences Flinders University Adelaide Australia

**Keywords:** social media, research use, knowledge translation, implementation science, conceptual framework

## Abstract

**Background:**

Social media has become widely used by individual researchers and professional organizations to translate research evidence into health care practice. Despite its increasing popularity, few social media initiatives consider the theoretical perspectives of how social media works as a knowledge translation strategy to affect research use.

**Objective:**

The purpose of this paper is to propose a conceptual framework to understand how social media works as a knowledge translation strategy for health care providers, policy makers, and patients to inform their health care decision-making.

**Methods:**

We developed this framework using an integrative approach that first involved reviewing 5 long-standing social media initiatives. We then drafted the initial framework using a deductive approach by referring to 5 theories on social media studies and knowledge translation. A total of 58 empirical studies on factors that influenced the use of social media and its messages and strategies for promoting the use of research evidence via social media were further integrated to substantiate and fine-tune our initial framework. Through an iterative process, we developed the Social Media for ImpLementing Evidence (*SMILE*) framework.

**Results:**

The *SMILE* framework has six key constructs: developers, messages and delivery strategies, recipients, context, triggers, and outcomes. For social media to effectively enable recipients to use research evidence in their decision-making, the framework proposes that social media content developers respond to target recipients’ needs and context and develop relevant messages and appropriate delivery strategies. The recipients’ use of social media messages is influenced by the virtual–technical, individual, organizational, and system contexts and can be activated by three types of triggers: sparks, facilitators, and signals.

**Conclusions:**

The *SMILE* framework maps the factors that are hypothesized to influence the use of social media messages by recipients and offers a heuristic device for social media content developers to create interventions for promoting the use of evidence in health care decision-making. Empirical studies are now needed to test the propositions of this framework.

## Introduction

### Social Media Use in Health Care

Social media has been extensively used worldwide to communicate health-related information. For example, in China, one-third of the users of the social media platform WeChat—which is widely used for instant messaging and social networking [1]—receive and read health information through the platform [2]. In the United States, 32% of social media users post messages about friends and family members’ health experiences on social media [3]. Health care professionals use social media to provide health information and answer medical questions [4], and patients and caregivers use social media for self-care and health literacy [5]. In health care research, social media platforms such as Twitter, Facebook, and YouTube are increasingly used for participant recruitment, intervention implementation, data mining and collection, and the sharing of research findings [6].

Social media, with its free access, interactive features, and widespread reach, has become increasingly used by individual researchers and professional organizations who wish to translate research evidence into health care practice. For example, the Joanna Briggs Institute (JBI) at Fudan University in China has been using WeChat to disseminate nursing evidence since 2014. In the first 2 years, their WeChat account reached 22,369 followers from 34 provinces in China [7,8]. The Cochrane Child Health groups in Canada and Portugal used social media strategies to disseminate child health evidence to health care providers, and within 6 months of initiating the strategy, their blog received 2555 visitors and 3967 page views, and their Twitter account gained 469 new followers from a geographically diverse population [9]. A social media initiative called *It Doesn’t Have to Hurt*, led by health care researchers in Canada, developed a short YouTube video on evidence-based strategies, such as distraction and using topical anesthetics for reducing procedural pain in children. Their video received 237,132 unique views from 182 countries 5 years after its launch, with patients and health care providers reporting strong acceptance and high intention to use the strategies [10]. The number of parents reporting the use of topical anesthetic creams to reduce pain increased from 18% to 63% after watching the video [11].

There has also been a surge in social media initiatives during the COVID-19 pandemic, which are aimed at helping health care professionals, patients, and the public better understand the coronavirus and cope with its impacts. Global evidence synthesis networks such as Cochrane, JBI, and Campbell Collaboration use social media to disseminate rapid review findings related to COVID-19. In China, the Beijing University of Chinese Medicine (BUCM) Cochrane Center, together with 20 evidence-based health care research teams and organizations, launched the *Fighting COVID-19 with Evidence* initiative. They collect urgent clinical questions about COVID-19 diagnosis, treatment, and nursing care through WeChat and share recommendations after a rapid search and synthesis of research evidence [12]. In England, the Center for Evidence-Based Medicine at Oxford University uses Twitter (@CebmOxford) to share COVID-19 relevant recommendations to a global audience. In Canada, the COVID-19 Evidence Network to support Decision-making initiative (@COVID_E_N_D) collects the best available evidence related to COVID-19 and shares this information on Twitter to support decision-making.

### Theoretical Understandings of Social Media as a Knowledge Translation Strategy

Despite its popularity, many researchers and organizational decision-makers upload research findings onto social media platforms without deliberately planning how to facilitate its use by recipients in policies, programs, or practices. In their systematic review, Webb et al [13] concluded that theory-based internet interventions had greater impacts on health behaviors than non–theory-based interventions, with interventions based on the *theory of planned behavior* having larger effects than those based on the *transtheoretical model* or *social cognitive theory.* However, despite these benefits, theoretical frameworks are rarely used to guide the development of social media interventions aimed at facilitating research use. In their systematic review, Arguel et al [14] only identified 15 experimental studies published between 2005 and 2016 that applied theoretical approaches to guide the development of social media interventions.

Ngai et al [15,16] classified 31 theories used in social media studies into three categories: personal behavior theories, social behavior theories, and mass communication theories. Personal behavior theories (eg, the *theory of planned behavior* and *technology acceptance model*) focus on personal factors that affect user behavior on social media. Social behavior theories (eg, *social capital theory* and *social cognitive theory*) identify key social factors that stimulate individuals to participate in collective actions on social media. Mass communication theories (eg, *parasocial interaction theory*) reveal the distinct characteristics of social communications that can assist in the use of social media for communication and marketing [15,16]. These theories provide valuable insights into social media’s role in behavior change; however, the following two limitations exist in fully understanding the research use process:

1. They only consider 1 of the 2 latent and indispensable layers of social media use: social media and messages. Recipients must first use social media before they can engage with messages (eg, the *technology acceptance model* emphasizes the platform, and the *social cognitive theory* and *theory of planned behavior* focus on the message). Theories that do not address both layers fail to fully explain the process of research use through social media.
2. They neglect multilevel contextual factors, such as the virtual–technical, organizational, and system contexts, particularly in relation to the features of the social media platform in shaping behavior. This may lead to the development of knowledge translation strategies solely from an individual perspective, without taking into account the contextual determinants that affect recipients’ behaviors.

These 2 limitations were partially addressed by Ritterband et al [17], who developed a *behavior change model for internet interventions*, which posited that website use was influenced by support, characteristics of the websites and users, and environmental factors. Behavior change from information on websites is then influenced by various mechanisms (eg, knowledge and motivation). This model has been used to guide the development and evaluation of internet interventions in health care [18,19]. Although not exactly the same, websites that allow for multiway interaction are normally considered to be social media [20,21], and the Ritterband et al [17] model has been used in the social media context [22]. It addresses the limitations of the aforementioned social media theories, as it considers the platform—which in this case is the website—and accounts for the multilayered contexts in shaping behavior, such as personal, professional, and community contexts, as well as the health care system [17]. However, the Ritterband et al model [17] does not make mechanisms of change explicit and presents a linear process for using the internet to change behavior when real-world practice is often complex [17].

Despite its extensive use for disseminating health care research evidence, social media is rarely used in a well-planned way with end users in mind, which largely limits its potential to bridge evidence–practice gaps and contribute to health care practices. Studies on the use of research evidence through social media are sparse [14]. Large theoretical gaps exist in understanding how social media interventions affect health care practices and decision-making. Unpacking the process by which social media works as a knowledge translation strategy is important to not only advance science but also inform interventions for improving health care practices and patient outcomes.

### Objective

The purpose of this paper is to propose a conceptual framework to understand how social media works as a knowledge translation strategy for health care providers, policy makers, and patients to inform their health care decision-making.

## Methods

We used a 3-step process based on the approach described by Meleis [23] to develop our conceptual framework. Meleis suggested that practice, theory, and research are important sources for patterning real-world phenomena and informing theory development [23]. Our approach was iterative and flexible and built a preliminary understanding of the process through which social media works for knowledge translation.

To get a sense of how they operate, we first reviewed five long-standing social media initiatives that have a large number of followers: the Fudan University JBI Center Nursing Evidence Dissemination Initiative (ie, Fudan JBI Initiative) [7,8,24], BUCM Cochrane Evidence Dissemination Initiative (ie, BUCM Cochrane Initiative) [25], *It Doesn’t Have to Hurt* initiative [10,26], *Be Sweet to Babies* initiative [27], and Translating Evidence in Child Health to Enhance Outcomes (ECHO) program [28]. For each social media initiative, we specifically reviewed the topics and interface of their social media channels (including format and structure of content); the number of readers, followers, and comments; intervals between posts; and the length of videos and papers published relating to each initiative.

Second, we drafted the initial framework using a deductive approach based on existing theories and our team members’ expertise in knowledge translation and social media. We primarily drew on five well-known and widely cited theories, frameworks, and models: *integrated Promoting Action on Research Implementation in Health Services (i-PARIHS*) [29]; *capability, opportunity, motivation, and behavior* (*COM-B*) [30]; *Fogg behavior model* [31]; *theory of innovation diffusion* [32]; and *behavior change model for Internet interventions* [17]. We built the basic structure of our framework based on the *i-PARIHS* *framework*, which argues that successful knowledge translation relies on the interactions among four constructs: innovation, recipients, context, and facilitation. In addition to the *i-PARIHS* constructs, we added one construct for social media content developers (hereafter referred to as *developers*) in recognition of the crucial role they play in ensuring that recipients get relevant and appropriate messages. We added the virtual–technical context to the 3-layer contexts described in *i-PARIHS* (ie, local, organizational, and external) to capture the unique features of social media platforms, which is substantiated by the *behavior change model for internet interventions* (described above). We also included three types of knowledge translation outcomes—conceptual, instrumental, and persuasive research use [33,34]—in recognition of the fact that not all evidence on social media was appropriate for practice or behavior change. Rather, we recognize that a large amount of social media evidence affects understanding, attitudes, or collective actions.

The other four theories and models were used to develop two further aspects of our framework: using social media and using the messages. In the first aspect, the four theories and models were employed to understand social media use from two main construct levels: recipients and the virtual–technical context. In the second aspect, derived from the *COM-B model* and the *Fogg behavior model*, we built subconstructs for the active ingredient of message use, named as *trigger* in our framework*.*

We then reviewed published papers that incorporated the 5 long-standing social media initiatives (described earlier) and used strategies such as citation tracking from the papers we reviewed. The forward citation search was conducted using Google Scholar, and the backward citation search was conducted by screening the reference lists. We also conducted a citation snowballing search using Google Scholar and consulted experts from the 5 social media initiatives and our team members to further locate relevant empirical studies. The studies we identified were primarily about factors that influenced people’s use of social media and its messages and strategies for promoting message use. We used the key findings of these studies to substantiate and fine-tune our initial framework. Through an iterative process, we went back and forth from the initial framework to social media initiatives, theories, and empirical studies and developed the Social Media for ImpLementing Evidence (*SMILE*) framework. *Implementation* in the *SMILE* framework refers to instrumental, conceptual, and persuasive knowledge translation.

## Results

### Overview

**Figure 1:**
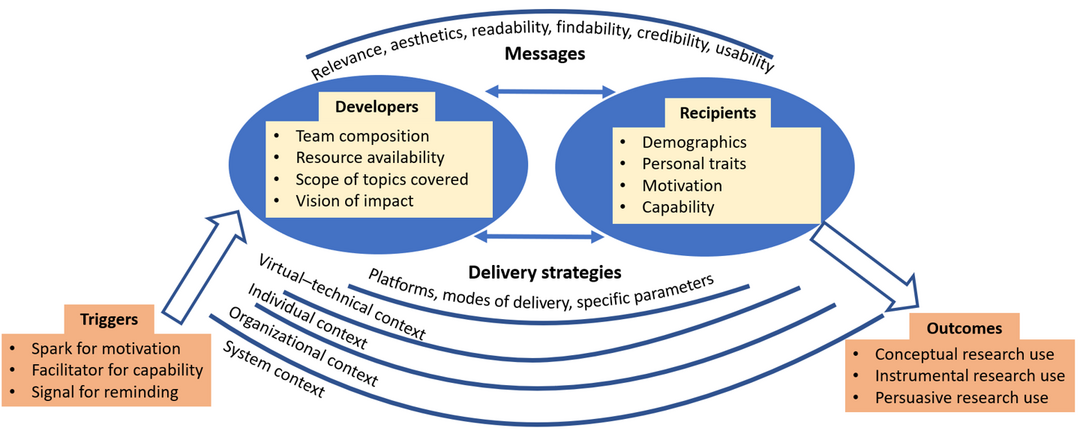
SMILE (Social Media for ImpLementing Evidence) framework.

Through a review of social media initiatives (n=5), theories (n=5), and empirical studies (n=58), including papers (15/58, 26%) relevant to the 5 social media initiatives [7,8,10,24,26,27,35-43] and papers (43/58, 74%) [9,13,15,44-83] from citation tracking, snowballing, or consultation, we developed the *SMILE* framework (Figure 1). Table 1 summarizes the key constructs and their supporting evidence. The *SMILE* framework provides a preliminary understanding of how social media can be used as a knowledge translation strategy to inform health care practices and decision-making. It has six key constructs: (1) developers, (2) messages and delivery strategies, (3) recipients, (4) context, (5) triggers, and (6) outcomes. For social media to enable recipients to use research evidence in their practice or decision-making, the framework proposes that developers respond to the needs and context of target recipients to develop relevant messages and appropriate delivery strategies. Recipients’ use of social media messages is influenced by the virtual–technical, individual, organizational, and system contexts and can be activated by different types of triggers, described as sparks, facilitators, and signals. Next, we describe the constructs of the *SMILE* framework.

**Table 1:** Key constructs in the Social Media for ImpLementing Evidence (SMILE) framework and the supporting evidence.

Constructs			Theory origins		Empirical studies		Social media initiatives
**Developers**			—^a^		[9,78]		Fudan JBI^b^ Initiative [7,8,24,84]; BUCM^c^ Cochrane Initiative [25]; *It Doesn’t Have to Hurt* initiative [10,26,85]; *Be Sweet to Babies* initiative [27]; ECHO^d^ [28,43]
	Team composition			[9]		Fudan JBI Initiative [7,8,24]; BUCM Cochrane Initiative [25]; *It Doesn’t Have to Hurt* initiative [85]; *Be Sweet to Babies* initiative [27]; ECHO [28]	
	Resource availability			[9]		Fudan JBI Initiative [7,8,24]; BUCM Cochrane Initiative [25]; *It Doesn’t Have to Hurt* initiative [10,26]; *Be Sweet to Babies* initiative [27]; ECHO [28]	
	Scope of topic covered			[9]		Fudan JBI Initiative [7,8,24]; BUCM Cochrane Initiative [25]; *It Doesn’t Have to Hurt* initiative [10,26]; *Be Sweet to Babies* initiative [27]; ECHO [28,43]	
	Vision of impact			[51,79]		Fudan JBI Initiative [84]; BUCM Cochrane Initiative [25]; *It Doesn’t Have to Hurt* initiative [10,26,85]; *Be Sweet to Babies* initiative [27]; ECHO [28,43]	
**Messages and delivery strategies**			*i-PARIHS*^e^*framework* (innovation) [29]		[51,57]		Fudan JBI Initiative [7,8,24]; BUCM Cochrane Initiative [25]; *It Doesn’t Have to Hurt* initiative [10,26]; *Be Sweet to Babies* initiative [27]; ECHO [28,43]
	Messages	*Behavior change model for internet interventions* (website) [17]		[44,47,49,52,54,56,58-63,80]		Fudan JBI Initiative [7,8,24]; BUCM Cochrane Initiative [25]; *It Doesn’t Have to Hurt* initiative [10,26]; *Be Sweet to Babies* initiative [27]; ECHO [28,43]	
	Delivery strategies	*Behavior change model for internet interventions* (website) [17]		[13,82,83]		Fudan JBI Initiative [7,8,24]; BUCM Cochrane Initiative [25]; *It Doesn’t Have to Hurt* initiative [10,26]; *Be Sweet to Babies* initiative [27]; ECHO [28,43]	
**Recipients**			*i-PARIHS framework* (recipients) [29]		[15,48,55,65]		—
	Demographics	*Behavior change model for internet interventions* (user characteristics [17]		[15,45,53,55]			
	Personal traits	*Behavior change model for internet interventions* (user characteristics) [17]		[46,64]			
	Motivation	*COM-B*^f^*model* (motivation and capability) [30]; *Fogg behavioral model* (motivation and capability) [31]		[48,50,55,65]			
	Capability	*COM-B model* (motivation and capability) [30]; *Fogg behavioral model* (motivation and capability) [31]		[48,50,55,65]			
**Context**			*i-PARIHS framework* (context) [29]		[81]		
	Virtual–technical context	*Behavior change model for internet interventions* (website) [17]; *theory of innovation diffusion* (innovation characteristics) [32]		[48,65,69,70,75,77]		Fudan JBI Initiative [7,8,24]; BUCM Cochrane Initiative [25]; *It Doesn’t Have to Hurt* initiative [10,26]; *Be Sweet to Babies* initiative [27,39,40]; ECHO [28]	
	Individual context	*Behavior change model for internet interventions* (environment) [17]; *COM-B model* (environment) [30]		[67,68]		Fudan JBI Initiative [8,24]; *It Doesn’t Have to Hurt* initiative [10,26]; *Be Sweet to Babies* initiative [39,40]	
	Organizational context	*Behavior change model for internet interventions* (environment) [17]; *COM-B model* (environment) [30]		—		*It Doesn’t Have to Hurt* initiative [10,26]; *Be Sweet to Babies* initiative [39,40]	
	System context	*Behavior change model for internet interventions* (environment) [17]; *COM-B model* (environment) [30]		[71]		*Be Sweet to Babies* initiative [39,40]	
**Triggers**			*i-PARIHS framework* (facilitation) [29]		[13,66,73,74]		—
	Spark for motivation	*Fogg behavioral model* (trigger) [31]; *COM-B model* (motivation and capability) [30]; *behavior change model for internet interventions* (support) [17]		[55,67,74,79]		*Be Sweet to Babies* initiatives [41]	
	Facilitator for capacity	*Fogg behavioral model* (trigger) [31]; *COM-B model* (motivation and capability) [30]; *behavior change model for internet interventions* (support) [17]		[13,72,76]		*Be Sweet to Babies* initiatives [27,42]	
	Signal for reminding	*Fogg behavioral model* (trigger) [31]; *COM-B model* (motivation and capability) [30]; *behavior change model for internet interventions* (support) [17]		[13,55,74]		—	
**Outcomes**			*i-PARIHS framework* (successful implementation) [29]		[33,34]		—
	Conceptual research use	—		—		*It Doesn’t Have to Hurt* initiative [10,26]; *Be Sweet to Babies* initiative [27,35-39];	
	Instrumental research use	—		[76]		*Be Sweet to Babies* initiative [41]	
	Persuasive research use	—		—		—	

^a^Data not available.

^b^JBI: Joanna Briggs Institute.

^c^BUCM: Beijing University of Chinese Medicine.

^d^ECHO: Evidence in Child Health to Enhance Outcomes.

^e^i-PARIHS: integrated Promoting Action on Research Implementation in Health Services.

^f^COM-B: capability, opportunity, motivation, and behavior.

### Developers

Developers are individuals, groups, and organizations responsible for the management of social media contents. Developer activities may include designing and periodic uploading of information, monitoring operations, collecting data on impact, and answering questions or comments from viewers. Developers can be health care researchers who produce research evidence and share it directly via social media for public access. Barton [78] proposed a new research-to-practice continuum where researchers not only disseminate research findings through traditional journal publications but also create multimedia messages and disseminate them to the public. Developers can also be intermediaries who serve as a link between research producers and end users by translating research evidence into user-friendly messages for dissemination on social media.

Although it might be simple for individuals to develop and upload research findings to social media, a fast, frugal, and *hope-the-change-happens* approach has limitations. One of the propositions embedded in the framework is that the composition of the development team, availability of resources, scope of topics, and vision of impact influence the development of relevant and appropriate social media interventions, thus affecting recipients’ engagement with and use of the messages.

We suggest bringing together a multidisciplinary collaborative team of health care professionals, target users, social media experts, and audiovisual technicians (eg, camera operators and video editors) to best support the development of social media interventions [9]. Health care professionals can assist with the identification of different types of evidence resources; target users can strengthen the relevance and accessibility of messages; social media experts can contribute to the operation of the platform; and audiovisual technicians can provide support when the team wants to deliver messages using videos or animations. For example, the *It Doesn’t Have to Hurt* initiative has built a large interdisciplinary collaborative team of researchers, trainees, patients, and other stakeholders to facilitate the stable operation of their social media program [10,26,85]. Similarly, the ECHO research program has created various videos, animations, and posters on child health with a multidisciplinary team [28]. With different knowledge, skills, and perspectives, the team can generate high-quality and influential social media products. The long-term collaborative approach can additionally promote the sustainability of these initiatives.

The availability of resources to develop and manage social media initiatives, such as time and budget, must be taken into consideration when planning it. In the *It Doesn’t Have to Hurt* initiative, it cost the team Can $15,000 (US $11,802) and considerable efforts to develop and promote their YouTube video, and the developers stated that financial and time costs could be a hindrance for individual researchers to undertake the work [10]. In their social media initiative to disseminate Cochrane Child Health evidence, Dyson et al [9] also found that the team invested enormous time and human resources in managing the platform. Therefore, we suggest that adequate time and budget be allocated to social media initiatives before their commencement.

The scope of topics covered is closely linked to the amount of time and resources invested. Some initiatives, such as the Fudan JBI Initiative [7,8,24] and BUCM Cochrane Initiative [25], have broad scopes that are open to a range of topics in nursing and medicine. Some initiatives focus only on specific topics; for example, the *It Doesn’t Have to Hurt* [10,26] and *Be Sweet to Babies* initiatives [27] target reducing procedural pain for children and infants, respectively. Other initiatives center on a certain field, such as the ECHO initiative, which covers common childhood conditions. The topics covered should be balanced with the consideration of practical issues. Dyson et al [9] suggested that starting from a specific content area and engaging with a stable social media community was more effective for developing a social media network.

It is also essential that the development team builds a shared vision of the impact they are looking to achieve and tracks the performance of their social media initiatives [51]. Building and sustaining a social media initiative is demanding work that requires collaboration and investment. An explicit team vision of the impact of social media can motivate the team to work toward a common goal. For example, since 2016, the Fudan JBI Initiative has openly shared its social media vision in its annual center report and at conferences [84]. Gates et al [79] also emphasized the importance of setting goals and tracking achievements after the evaluation of their social media initiative.

In the *SMILE* framework*,* we propose that the engagement of a multidisciplinary team, time, and resource investments are essential for developing relevant and appropriate social media interventions to influence research use. Developers should balance the topics covered with practical considerations and create a shared vision of the goals of their social media initiatives.

### Messages and Delivery Strategies

#### Overview

The second construct in the *SMILE* framework is messages and delivery strategies. Developers should respond to recipients’ needs and their context to create messages and delivery strategies. Through a systematic literature review, Schein et al [57] observed that collaborating with target users to create social media interventions contributed to heightened authenticity of messages and improved trust in developers. Korda and Itani [51] suggested that social media messages should account for user characteristics and information preferences and should be customized through an iterative interaction with target users. On the basis of 4 years of experience in social media operations, the Fudan JBI Initiative recommended that developers could improve the usability and uptake of research evidence on WeChat through the full use of WeChat’s interactive functions to capture users’ needs [7,24].

#### Messages

To date, a limited number of studies have investigated the attributes of social media messages that influence its uptake, despite the development of tools and models to assess the quality of web-based information [44,47,49,54,59,61-63,80]. On the basis of the content of these tools and models, as well as the unique features of social media platforms, we posit six interrelated attributes that influence the uptake of social media messages: relevance, aesthetics, readability, findability, credibility, and usability.

A *relevant* message is directly related, connected, or pertinent to target users. The more relevant messages are to the target users, the higher their level of engagement and the likelihood of being used. In their systematic review, Schubart et al [58] concluded that internet interventions that addressed the primary concerns of patients with chronic health conditions were the most successful.

An *aesthetic* message is characterized by the artistic design and visual appeal of the social media content; for instance, the layout of content, color and size of words, and graphics [17]. A first impression is made after a brief glimpse of the format and structure of content, and a user will quickly decide whether to stay on it or leave [56]. For example, ECHO uses art-based approaches, such as animations and e-books, to disseminate child health evidence on social media [43]. As many social media platforms impose restrictions on the design and presentation of messages, flexibility with visual appeal is often limited. For example, Twitter only allows 140 characters and 4 pictures per tweet.

A *readable* message is easy to follow. The US National Institutes of Health recommend that the readability of content on websites be at the sixth- to eighth-grade level [44]. Readability also encompasses accessibility and understandability. Health information that is hard to read will be hard to understand and therefore remain inaccessible, particularly for people with low health literacy [52]. For example, the *It Doesn’t Have to Hurt* initiative developed YouTube video storyboards and scripts in collaboration with a communication company, which was further verified by parents for its readability [10,26].

The messages must also be *findable*, meaning that they are easy to locate. Search boxes, navigation menus, and links are likely to improve the findability of health information on social media [62,63]. Both the Fudan JBI Initiative [7] and BUCM Cochrane Initiative used the navigation function in WeChat to organize and categorize the evidence sources, which allowed users to easily locate the specific evidence item they wanted.

A *credible* message refers to the trustworthiness of the message and is described as accurate, believable, and factual [59,63,80]. The *Journal of the American Medical Association* considers four elements to judge the credibility of medical information on the internet: currency of information; declaration of authorship; presentation of a list of references; and the disclosure of any conflicts of interest, funding, or sponsorship [60].

Finally, the *usability* of a message is the extent to which it can be actionable in practice. For the purpose of affecting research use, clear behavioral recommendations or prescriptions within the message can promote its usability [44]. Together, the six attributes of relevance, aesthetics, readability, findability, credibility, and usability influence the use of a social media message in practice.

#### Delivery Strategies

Delivery strategies are the ways through which social media messages are conveyed to recipients. We conceptualize them as comprising three distinct layers: the social media platforms, modes of delivery, and specific parameters. One of the first decisions that developers need to make is which social media platform to use. Although social media platforms have burgeoned in recent years, only a few are popular for disseminating health care information, such as Facebook, YouTube, and Twitter in Western countries and WeChat and Weibo in Asia. Messages are delivered on social media platforms through different modes of delivery, such as text, infographics, videos, audios, animations, vignettes, testimonials, and stories [17]. The modes of delivery differ in their impact on users’ engagement with the messages, and research has found that visual abstracts attract a significantly greater number of engagements than basic texts [82,83]. Webb et al [13] conducted a systematic review in which they classified the modes of delivery of internet-based behavior change interventions into three types: automated functions (eg, automated tailored feedback), communicative functions (eg, access to an adviser to request advice), and the use of supplementary modes (eg, SMS text message). It should be noted that the options for the mode of delivery vary for different social media platforms. The specific parameters of the delivery strategy are the characteristics of the mode of delivery, such as the length of videos, size, color and limits of words, frequency, and interval of message uploading. In the 5 initiatives we reviewed, all used a variety of social media platforms such as WeChat, YouTube, and Twitter. In addition, they used diversified modes of delivery, such as videos, podcasts, animations, stories, and texts, to deliver their social media messages.

Overall, the attributes of messages and delivery strategies affect the reach and successful use of messages by people and are a key construct in the *SMILE* framework. The 6 attributes of messages and the 3 layers of delivery strategies should be considered during the social media content development process to promote the likelihood of message use.

### Recipients

Recipients are the target audience of social media messages and have the potential to direct, influence, or be affected by messages. In our framework, we consider health care providers, policy makers, and health care consumers as recipients. We also propose that using social media messages in health care decision-making involves two distinct, interconnected layers: using the social media and then using the message. It is a prerequisite for recipients to first accept and use the social media before they can engage with the messages. We distinguish between these 2 layers and consider the factors that influence each layer separately. We contend that the characteristics of recipients and the virtual–technical context are the two main domains that influence people’s use of social media, and the individual, organizational, and system contextual domains shape the message use.

Together with frameworks from the social media and technology research field [15,48,55,65], the *i-PARIHS framework* [29]*, behavior change model for internet interventions* [17], *COM-B model* [30]*,* and *Fogg behavior model* [31] have provided valuable insights into the characteristics of recipients that influence social media use. On the Basis of their theoretical constructs, four aspects of recipients’ characteristics were incorporated into our framework: demographics, personal traits, motivation, and capability.

Demographics include age, gender, geography, socioeconomic status, ethnicity, and lifestyles [15,17,55]. Large quantities of research data from Twitter and Facebook revealed differences in social media use by gender, ethnicity, and geography [45,53]. Personal traits of openness, conscientiousness, extraversion, agreeableness, and neuroticism—rooted in genetics—are perceived as one of the fundamental theories that explain personal behavior [15]. They are closely associated with social media use [64]. In a national survey in the United States, Correa et al [46] found that although extraversion and openness were positively related to social media use, emotional stability—a central measure of neuroticism—was a negative predictor. These findings differed by gender and age [46].

Motivation and capability are 2 summative characteristics of social media recipients that the *SMILE* framework identifies as affecting social media use. These characteristics are based on the *Fogg behavior model* [31] and the *COM-B model* [30]. Within motivation, perceived needs [65], attitude [50], intention [48,50,55], self-efficacy [17], and goals [55] are factors motivating individuals to use social media. Within capability [48,50], knowledge and skills [17,31] enable individuals to use social media. Together, all four characteristics of recipients (demographics, personal traits, motivation, and capability) are determinants affecting social media use in the *SMILE* framework.

### Context

#### Overview

In the *SMILE* framework, context is defined as “a set of characteristics and circumstances that consist of active and unique factors that surround the implementation... (It) interacts, influences, modifies and facilitates or constrains the intervention and its implementation” [81]. We identify four interrelated layers of contextual factors that influence social media use and further message use: virtual–technical, individual, organizational, and system contexts.

#### Virtual–Technical Context

The virtual–technical context is the context surrounding the social media platform. Dawot and Ibrahim [69] summarized its composition into three core elements: individual-level, conversation-level, and community-level elements. Through a systematic review, Elaheebocus et al [70] created a taxonomy of social media features that included identity representation, communication, peer grouping, data sharing, competition, activity data viewing, and web-based social networks.

We posit that seven characteristics of the platform influence social media use: relative advantage, complexity, observability, compatibility, usefulness, interactivity, and playfulness [48,65,75,77]. Relative advantage, complexity, observability, and compatibility originate from the *theory of innovation diffusion* [32] and are all considered important factors influencing social media use [65]. *Relative advantage* is the degree to which one social media platform is perceived to be better than other alternatives. *Complexity* is the extent to which social media is perceived as being difficult to use. *Observability* is the degree to which the benefits of social media use are visible to others. *Compatibility* is the degree to which social media is perceived as consistent with the existing values, past experiences, and needs of potential users [65]. Each of these factors is positively associated with social media use, except for complexity [75]; the more complex the social media is perceived, the lower the level of engagement by users. *Usefulness* is the degree to which social media can directly or indirectly benefit individual performance. Data show that usefulness can predict up to 62% of the intention to use social media [48]. *Interactivity* is the degree to which social media enables 2-way communication rather than 1-way transmission or distribution of information. Multiple research studies have demonstrated the positive effects of interactivity on social media use [75,77]. *Playfulness* is the hedonic value of social media and can influence the perceived usefulness and direct use of social media [48]. In the 5 social media initiatives included for developing the *SMILE* framework, all of them use popular platforms that contain these 7 characteristics, attesting to their importance. We posit that all 7 aspects of the platform in the virtual–technical context affect social media use.

#### Individual Context

The context of an individual plays a crucial role in shaping one’s behavior of message use. Brouwer et al [67,68] found that being motivated to visit the web-based intervention, being curious about the content, and perceiving the web-based intervention as personally relevant were important influencers for participants to engage with the web-based intervention. In a qualitative and a cross-sectional study conducted by Hu et al [39,40] to understand the barriers of implementing the *Be Sweet to Babies* pediatric pain management strategies in China, they found that insufficient knowledge, beliefs, and self-efficacy of health care providers were common individual-level barriers hindering the implementation of social media messages in clinical practices by nurses.

#### Organizational Context

Organizational context is considered an indispensable layer of the context affecting one’s use of a social media message in practice. In the *Be Sweet to Babies* initiative, the hierarchical managerial system, low authority of nurses, and staff shortage were factors impeding nurses from changing their practice and incorporating the evidence in China [40]. In the *It Doesn’t Have to Hurt* initiative, researchers found that the cost for using topical anesthetic cream [10] and the unit routines of disallowing parental presence during painful procedures [26] hindered the implementation of pain management strategies for children. The Fudan JBI Initiative also stated explicitly in every WeChat post that users should consider the local context to determine the appropriateness of implementing the evidence.

#### System Context

People’s use of social media messages in health care practices is also influenced by the broader system context, namely the social, political, economic, and cultural environment. From a social perspective, one study found that popular opinion leaders on the internet played a positive role in changing sexual behaviors among men who have sex with men [71]. Some countries impose restrictions at the judicial level on accessing certain social media, which may be attributed to ideological, political, or economic reasons. Culturally, Hu et al [40] found that the negatively escalating relationships between patients and health care professionals in China made nurses reluctant to introduce the *Be Sweet to Babies* pain management strategies, despite a strong evidence base for the practices.

As illustrated above, we have made distinctions between the four types of contexts that influence social media and its message use in the *SMILE* framework. Specifically, the virtual–technical context concerns the determinants of social media use; the individual, organizational, and system contexts are considered as the micro-, meso-, and macro-level factors shaping message use.

### Triggers

#### Overview

The concept of the *trigger* in the *SMILE* framework describes the strategies adopted to activate social media message use. On the basis of the *i-PARIHS framework* [29], for social media to be effective in facilitating research use, there needs to be an active ingredient to energize the message implementation process, in addition to having relevant messages. The trigger is derived from the *Fogg behavioral model* [31] and includes behavior change techniques (active triggers) or events (passive triggers) that activate a recipient to use social media messages. One behavior change technique, as an active trigger, is an “observable, replicable, and irreducible component of an intervention designed to alter or redirect causal processes that regulate behavior” [73]. Michie et al [73] created a behavior change technique taxonomy to standardize the reporting of the active content of behavior change interventions. These techniques have been widely adopted in social media interventions. Webb et al [13] found, in their systematic review, that internet interventions that incorporated more behavior change techniques had larger effects than interventions that incorporated fewer techniques. In a systematic review of the characteristics of internet-delivered healthy lifestyle promotion interventions, Brouwer et al [66] reported that feedback, interactive elements, and email or phone contact were the most commonly used techniques. In a recent systematic review in 2020, Simeon et al [74] conducted a detailed analysis of the behavior change techniques used in social media interventions. They found that 46 techniques had been used in the identified 71 studies. An event, as a passive trigger, is an emergent, unexpected, or accidental incident that pushes recipients to use social media messages in a passive way. These events require people to think and act in alternative ways, and social media provides relevant information to perform an alternative behavior. Fogg [31] classified triggers into three different types in persuasive technology design: sparks, facilitators, and signals. We adopted these 3 types of triggers and enriched their connotations in our framework, as discussed in the following sections.

#### Spark for Motivation

A spark is a trigger that motivates recipients to use a message. It can be used when recipients’ motivation to use a social media message is low or needs to be further enhanced. Developers can apply various behavior change techniques such as problem solving, feedback and monitoring, and social support and reward [55,74] to help activate behavior change. For example, Modanloo [41] used motivational interviewing to improve parents’ use of *Be Sweet to Babies* pain management strategies for infants during vaccination. Although no significant differences were found between the 2 comparative groups, approximately all participants used at least one strategy in the vaccination [41]. Through a Delphi study approach, Brouwer et al [67] identified that two behavior change techniques—the provision of tailored feedback on behavior and credible information source [73]—were related to an extended engagement with internet interventions. After implementing their social media initiative, Gates et al [79] suggested that web-based opinion leaders’ endorsements would be a promising strategy for motivating recipients to use the messages.

#### Facilitator for Capability

A facilitator is a trigger that improves recipients’ capability to use social media messages, such as knowledge and skills. Social media interventions that incorporate different behavior change techniques, such as instructions on behavior performance and demonstrating the behavior [73], are likely to improve the capability of recipients. In the Clinical Excellence Through Social Media trial, Tunnecliff et al [76] linked every tendon management practice point on Twitter and Facebook to supplementary information to enhance the knowledge of recipients. Webb et al [13] found that the use of communicative functions within internet interventions to provide access to and schedule contacts with an adviser could have a small to medium effect on behavior. Developers made full use of the visualization function of a YouTube video in the *Be Sweet to Babies* initiative to demonstrate pain management techniques and help the recipients build skills [27]. Watching this video doubled the chance of using an analgesic strategy and increased breastfeeding 1.5 times and skin-to-skin care 4.6 times by parents in a nonrandomized pragmatic trial in Brazil [42].

#### Signal for Reminding

A signal indicates or reminds recipients of social media messages. This type of trigger is useful when recipients need external reminders to use messages or, in other cases, when events emerge, and the developers want to push recipients to use the messages, such as wearing masks during the COVID-19 pandemic. The signal can be an active prompt or cue in the form of an SMS text message delivered by developers [31,55]. Among the 71 included studies in the systematic review by Simeon et al [74], 10 studies reported the use of prompts or cues as a behavior change technique in self-directed social media programs. Webb et al [13] also found that SMS text messages were highly effective for behavior change in internet interventions when they provided cues to action. A signal, on the other hand, is an event that is emergent, accidental, or unexpected such as an adverse event that happened on a unit, a new health care regulation or policy, or a global pandemic. These events remind people of the relevant resources on social media platforms that can help tackle the situation. Together, the *SMILE* framework proposes sparks, facilitators, and signals as triggers to activate recipients to use social media messages.

### Outcomes

In the *SMILE* framework, we specify the knowledge translation outcome as research use, which is a multidimensional concept that involves conceptual, instrumental, and persuasive use of research findings [33,34]. Conceptual research use refers to using research evidence to change the levels of knowledge, understanding, or attitude of a recipient. Both the *It Doesn’t Have to Hurt* initiative [10,26] and *Be Sweet to Babies* initiative [27,35-39] have demonstrated that when recipients receive relevant and appropriate messages on social media that respond to their needs and context, they are highly likely to improve conceptual research use. Instrumental research use involves the direct application of research evidence in practice to change behavior. Modanloo et al [41] and Tunnecliff et al [76] have shown, in their randomized controlled trials, that different types of triggers, such as sparks or facilitators, are essential for the active uptake of research evidence and behavior change by recipients. Persuasive research use refers to using research evidence as a political or persuasive tool to justify an action, attain power, or achieve goals [33,34]. One of the most typical examples is the #WearingMasks social media campaign during COVID-19, which has made a huge impact on public behavior and government policy making.

## Discussion

### Principal Findings

In this paper, we present the *SMILE* framework, which is based on a review of 5 social media initiatives, 5 theories, and 58 empirical studies. The framework provides a preliminary understanding of how social media works as a knowledge translation strategy for health care providers, policy makers, and patients to inform their health care decision-making.

The *SMILE* framework has implications for research by offering a heuristic device for the development of social media interventions to promote evidence use. We suggest that it be used in combination with process frameworks, which provide step-by-step guidance on implementing web-based knowledge translation interventions [86] or evaluation frameworks to evaluate the multilevel outcomes and impacts of social media interventions [54].

### Implications for Social Media Strategy Development

On the basis of this framework, we offer several suggestions for researchers and organizations who intend to use social media to promote research use. First, in the preparation stage, it is important for developers to assess their readiness to start a social media initiative. Some probing questions may be considered during this stage, such as is there an explicit topic to be covered? Does the team have enough time, resources, and expertise to develop the intervention and monitor the operation?

Once the infrastructure has been built for the social media initiative, the team begins developing a message and delivery strategy. Developers should recognize target users’ needs and their context and, if possible, engage them in the development process. The six attributes of messages (ie, relevance, aesthetics, readability, findability, credibility, and usability) and three dimensions of delivery strategies (ie, social media platform, mode of delivery, and specific parameters) need to be taken into account when creating the social media interventions.

The team can then start the activation stage, where they make efforts to embed triggers into the social media delivery mechanisms for recipients to use the messages. Developers may interact with multilevel stakeholders and investigate the enablers of and barriers to recipients’ use of the messages. By tailoring behavior change techniques to identified barriers and enablers, the development team can develop a social media strategy that has the greatest potential to affect message use in practice.

### Acknowledgment of Complexity Within This Framework

We fully acknowledge the complexity of developing and implementing social media interventions and incorporate the notion of complexity within this framework in several ways. As information and communication sciences are fast-growing fields, new features and functions for social media platforms are continually emerging. Consequently, the approaches to developing messages and delivery strategies may become more diversified as technology advances. The dynamic interactions between constructs within the *SMILE* framework, such as the interaction between developers, recipients, and their situated contexts, make it challenging to undertake firm predictions [87,88]. Developers should immerse themselves in the human–social media system and capture underlining interactive patterns to inform the development of the most relevant and targeted activating techniques.

We also acknowledge the nonlinear aspect of the social media implementation processes, in which different levels of context influence and shape behavior. Promoting research use through social media is not a linear, straightforward process, and constant adaptations should be expected and embraced to optimize interventions. Finally, as each construct within this framework does not have a fixed and predetermined effect, and the interactions between constructs are dynamic and complex, we recognize that the framework has not been empirically validated and may not reveal all of the mechanisms at play for social media to influence research use. Nevertheless, the framework is based on current empirical evidence and well-recognized theories to provide plausible explanations for the successes and failures of social media interventions. Overall, the framework explicates the complexities of using social media in real-world practice and elucidates the key domains that developers, recipients, and researchers should attend to when developing or evaluating social media interventions.

As Maloney et al [72] suggested, “rather than looking at whether or not social media is effective for health professional education, it may be time to look at how various modalities can be optimized, both in terms of how the messages are delivered and how learners can be supported to engage.” Using social media to disseminate research evidence has become such an inexorable global trend that researchers should go beyond the investigation of the effectiveness of social media interventions and delve into the theoretical field on how to make it effective. The next stage of our project will be to test and refine the *SMILE* framework through a realist methodology that unpacks the mechanisms of how and under what circumstances social media works as a knowledge translation strategy for health care professionals to improve the delivery of research-based care.

### Limitations

The *SMILE* framework and its development process have 2 limitations. First, because of the multiple interactive components involved in developing and using social media for knowledge translation, as well as the massive amount of literature available from the relevant disciplinary fields, it was challenging to retrieve all pertinent theories and studies using a full systematic review approach. Instead, we used a targeted and flexible approach to select studies that allowed us to prioritize articles based on our framework’s development needs. It is possible that we missed some research and embedded our own values into the propositions by using this approach; thus, our next step is to test and refine the framework. Second, as the use of social media for knowledge translation in real-world practice is still in its infancy, we could not locate studies that captured all the *SMILE* framework’s propositions. More empirical studies of social media initiatives are needed to test the propositions of this framework.

### Conclusions

In this paper, we propose the *SMILE* framework based on a review of social media initiatives, theories, and empirical studies as a preliminary understanding of how social media works as a knowledge translation strategy in health care decision-making. We provide a detailed description of each construct in the framework and offer suggestions for researchers and developers who intend to develop social media initiatives and interventions. For social media to be effective in enabling recipients to use research evidence in their practice decision-making, the *SMILE* framework purports that developers respond to target recipients’ needs and context to develop relevant social media messages and appropriate delivery strategies. Recipients’ use of messages is influenced by the virtual–technical, individual, organizational, and system contexts and can be activated by three types of triggers: sparks, facilitators, and signals. The *SMILE* framework maps the factors that are hypothesized to influence recipients’ social media message use and offers a heuristic device for social media developers and researchers to develop social media interventions. More empirical studies and social media initiatives are needed to test the propositions of the *SMILE* framework.

## Abbreviations

BUCM: Beijing University of Chinese Medicine
COM-B: capability, opportunity, motivation, and behavior
ECHO: Evidence in Child Health to Enhance Outcomes
i-PARIHS: integrated Promoting Action on Research Implementation in Health Services
JBI: Joanna Briggs Institute
SMILE: Social Media for Implementing Evidence

## References

[1] Sun M, Yang L, Chen W, Luo H, Zheng K, Zhang Y, Lian T, Yang Y, Ni J (2021). Current status of official WeChat accounts for public health education. J Public Health (Oxf).

[2] Zhang X, Wen D, Liang J, Lei J (2017). How the public uses social media wechat to obtain health information in China: a survey study. BMC Med Inform Decis Mak.

[3] Warden C Liquid Lock.

[4] Moorhead SA, Hazlett DE, Harrison L, Carroll JK, Irwin A, Hoving C (2013). A new dimension of health care: systematic review of the uses, benefits, and limitations of social media for health communication. J Med Internet Res.

[5] Hamm MP, Chisholm A, Shulhan J, Milne A, Scott SD, Given LM, Hartling L (2013). Social media use among patients and caregivers: a scoping review. BMJ Open.

[6] Dol J, Tutelman PR, Chambers CT, Barwick M, Drake EK, Parker JA, Parker R, Benchimol EI, George RB, Witteman HO (2019). Health researchers' use of social media: scoping review. J Med Internet Res.

[7] Zhu Z, Xing W, Yan H, Zhou Y, Ying G, Cheng L (2017). Construction and effect evaluation of platform for evidence dissemination. Chin J Nurs.

[8] Zhu Z, Xing W, Hu Y, Zhou Y, Gu Y (2018). Improving evidence dissemination and accessibility through a mobile-based resource platform. J Med Syst.

[9] Dyson MP, Newton AS, Shave K, Featherstone RM, Thomson D, Wingert A, Fernandes RM, Hartling L (2017). Social media for the dissemination of Cochrane child health evidence: evaluation study. J Med Internet Res.

[10] Chambers CT, Dol J, Parker JA, Caes L, Birnie KA, Taddio A, Campbell-Yeo M, Halperin SA, Langille J (2020). Implementation effectiveness of a parent-directed YouTube video ("It doesn't have to hurt") on evidence-based strategies to manage needle pain: descriptive survey study. JMIR Pediatr Parent.

[11] #ItDoesntHaveToHurt: making a difference for children---science-media partnership harnesses social media to connect with parents and mobilize evidence on children's pain. Canadian Institutes of Health Research.

[12] Evidence-based Mutual Aid Action Against Pandemic: You Ask, We Answer. Beijing University of Chinese Medicine Cochrane Center.

[13] Webb TL, Joseph J, Yardley L, Michie S (2010). Using the internet to promote health behavior change: a systematic review and meta-analysis of the impact of theoretical basis, use of behavior change techniques, and mode of delivery on efficacy. J Med Internet Res.

[14] Arguel A, Perez-Concha O, Li SY, Lau AY (2018). Theoretical approaches of online social network interventions and implications for behavioral change: a systematic review. J Eval Clin Pract.

[15] Ngai EW, Tao SS, Moon KK (2015). Social media research: theories, constructs, and conceptual frameworks. Int J Inf Manag.

[16] Ngai EW, Moon KK, Lam S, Chin ES, Tao SS (2015). Social media models, technologies, and applications: an academic review and case study. Industr Manag Data Syst.

[17] Ritterband LM, Thorndike FP, Cox DJ, Kovatchev BP, Gonder-Frederick LA (2009). A behavior change model for internet interventions. Ann Behav Med.

[18] Ritterband LM, Bailey ET, Thorndike FP, Lord HR, Farrell-Carnahan L, Baum LD (2012). Initial evaluation of an internet intervention to improve the sleep of cancer survivors with insomnia. Psychooncology.

[19] Corkum PV, Reid GJ, Hall WA, Godbout R, Stremler R, Weiss SK, Gruber R, Witmans M, Chambers CT, Begum EA, Andreou P, Rigney G (2018). Evaluation of an internet-based behavioral intervention to improve psychosocial health outcomes in children with insomnia (better nights, better days): protocol for a randomized controlled trial. JMIR Res Protoc.

[20] Kaplan AM, Haenlein M (2010). Users of the world, unite! The challenges and opportunities of social media. Business Horizons.

[21] Petkovic J, Duench S, Trawin J, Dewidar O, Pardo Pardo J, Simeon R, DesMeules M, Gagnon D, Hatcher Roberts J, Hossain A, Pottie K, Rader T, Tugwell P, Yoganathan M, Presseau J, Welch V (2021). Behavioural interventions delivered through interactive social media for health behaviour change, health outcomes, and health equity in the adult population. Cochrane Database Syst Rev.

[22] Shaw C (2014). Implementing an online social network for health communication. The University of New Mexico UNM Digital Repository.

[23] Meleis A (2011). Theoretical Nursing: Development and Progress.

[24] Zhu Z, Xing W, Hu Y, Zhou Y, Gu Y, Cheng L (2017). The construction of evidence dissemination platform based on mobile terminals and the status of the consumers' demand and experience. J Nurs Train.

[25] Using social media platforms to disseminate Cochrane evidence in China. Cochrane Community.

[26] Campbell-Yeo M, Dol J, Disher T, Benoit B, Chambers CT, Sheffield K, Boates T, Harrison D, Hewitt B, Jangaard K, Stinson J, Taddio A, Parker JA, Caddell K (2017). The power of a parent's touch: evaluation of reach and impact of a targeted evidence-based YouTube video. J Perinat Neonatal Nurs.

[27] Harrison D, Wilding J, Bowman A, Fuller A, Nicholls SG, Pound CM, Reszel J, Sampson M (2016). Using YouTube to disseminate effective vaccination pain treatment for babies. PLoS One.

[28] Translating evidence in child health to enhance outcomes. EchoKT.

[29] Harvey G, Kitson A (2016). PARIHS revisited: from heuristic to integrated framework for the successful implementation of knowledge into practice. Implement Sci.

[30] Michie S, van Stralen MM, West R (2011). The behaviour change wheel: a new method for characterising and designing behaviour change interventions. Implement Sci.

[31] Fogg B (2009). A behavior model for persuasive design. Proceedings of the 4th International Conference on Persuasive Technology.

[32] Rogers E (2003). Diffusion of Innovations, 5th Edition.

[33] Graham ID, Logan J, Harrison MB, Straus SE, Tetroe J, Caswell W, Robinson N (2006). Lost in knowledge translation: time for a map?. J Contin Educ Health Prof.

[34] Straus S, Tetroe J, Graham I (2009). Knowledge Translation in Health Care: Moving from Evidence to Practice.

[35] Harrison D, Larocque C, Reszel J, Harrold J, Aubertin C (2017). Be sweet to babies during painful procedures: a pilot evaluation of a parent-targeted video. Adv Neonatal Care.

[36] Vieira AC, Bueno M, Harrison D (2020). “Be sweet to babies”: use of Facebook as a method of knowledge dissemination and data collection in the reduction of neonatal pain. Paediatric Neo Pain.

[37] Bueno M, Costa RN, de Camargo PP, Costa T, Harrison D (2018). Evaluation of a parent-targeted video in Portuguese to improve pain management practices in neonates. J Clin Nurs.

[38] Almeida HC, Candido LK, Harrison D, Bueno M (2018). Be Sweet to Babies: evaluation of an instructional video on neonatal pain management by nurses. Rev Esc Enferm USP.

[39] Hu J, Gifford W, Zhou Y, Zhang Q, Harrison D (2021). Nurses' perspectives on pain management practices during newborn blood sampling in China. J Neonatal Nurs.

[40] Hu J, Ruan H, Li Q, Gifford W, Zhou Y, Yu L, Harrison D (2020). Barriers and facilitators to effective procedural pain treatments for pediatric patients in the Chinese context: a qualitative descriptive study. J Pediatr Nurs.

[41] Modanloo S, Dunn S, Stacey D, Harrison D (2021). The feasibility, acceptability and preliminary efficacy of parent-targeted interventions in vaccination pain management of infants: a pilot randomized control trial (RCT). Pain Manag.

[42] Korki de Candido L, Harrison D, Ramallo Veríssimo MD, Bueno M (2020). Effectiveness of a parent‐targeted video on neonatal pain management: nonrandomized pragmatic trial. Paediatric Neo Pain.

[43] Reid K, Hartling L, Ali S, Le A, Norris A, Scott SD (2017). Development and usability evaluation of an art and narrative-based knowledge translation tool for parents with a child with pediatric chronic pain: multi-method study. J Med Internet Res.

[44] Abdel-Wahab N, Rai D, Siddhanamatha H, Dodeja A, Suarez-Almazor ME, Lopez-Olivo MA (2019). A comprehensive scoping review to identify standards for the development of health information resources on the internet. PLoS One.

[45] Chang J, Rosenn I, Backstrom L, Marlow C (2010). ePluribus: ethnicity on social networks. Proceedings of the Fourth International AAAI Conference on Weblogs and Social Media.

[46] Correa T, Hinsley AW, de Zúñiga HG (2010). Who interacts on the web?: the intersection of users’ personality and social media use. Comput Human Behav.

[47] Dubowicz A, Schulz PJ (2015). Medical information on the internet: a tool for measuring consumer perception of quality aspects. Interact J Med Res.

[48] Rauniar R, Rawski G, Yang J, Johnson B (2014). Technology acceptance model (TAM) and social media usage: an empirical study on Facebook. J Ent Info Management.

[49] Eysenbach G, Powell J, Kuss O, Sa E (2002). Empirical studies assessing the quality of health information for consumers on the world wide web: a systematic review. JAMA.

[50] Hazzam J, Lahrech A (2018). Health care professionals' social media behavior and the underlying factors of social media adoption and use: quantitative study. J Med Internet Res.

[51] Korda H, Itani Z (2013). Harnessing social media for health promotion and behavior change. Health Promot Pract.

[52] McInnes N, Haglund BJ (2011). Readability of online health information: implications for health literacy. Inform Health Soc Care.

[53] Mislove A, Lehmann S, Ahn Y, Onnela J, Rosenquist J (2011). Understanding the demographics of Twitter users. Proceedings of the Fifth International Conference on Weblogs and Social Media.

[54] O'Grady L, Witteman H, Bender JL, Urowitz S, Wiljer D, Jadad AR (2009). Measuring the impact of a moving target: towards a dynamic framework for evaluating collaborative adaptive interactive technologies. J Med Internet Res.

[55] Oinas-Kukkonen H, Harjumaa M (2009). Persuasive systems design: key issues, process model, and system features. Commun Assoc Inf Syst.

[56] Robins D, Holmes J (2008). Aesthetics and credibility in web site design. Inf Process Manag.

[57] Schein R, Wilson K, Keelan J Literature review on effectiveness of the use of social media: a report for Peel public health. Region of Peel.

[58] Schubart JR, Stuckey HL, Ganeshamoorthy A, Sciamanna CN (2011). Chronic health conditions and internet behavioral interventions: a review of factors to enhance user engagement. Comput Inform Nurs.

[59] Short CE, Gelder C, Binnewerg L, McIntosh M, Turnbull D (2018). Examining the accessibility of high-quality physical activity behaviour change support freely available online for men with prostate cancer. J Cancer Surviv.

[60] Silberg WM, Lundberg GD, Musacchio RA (1997). Assessing, controlling, and assuring the quality of medical information on the internet: caveant lector et viewor--Let the reader and viewer beware. JAMA.

[61] Stoyanov SR, Hides L, Kavanagh DJ, Zelenko O, Tjondronegoro D, Mani M (2015). Mobile app rating scale: a new tool for assessing the quality of health mobile apps. JMIR Mhealth Uhealth.

[62] Sun Y, Zhang Y, Gwizdka J, Trace CB (2019). Consumer evaluation of the quality of online health information: systematic literature review of relevant criteria and indicators. J Med Internet Res.

[63] Zhang Y, Sun Y, Xie B (2015). Quality of health information for consumers on the web: a systematic review of indicators, criteria, tools, and evaluation results. J Assn Inf Sci Tec.

[64] Zhong B, Hardin M, Sun T (2011). Less effortful thinking leads to more social networking? The associations between the use of social network sites and personality traits. Comput Human Behav.

[65] Zolkepli IA, Kamarulzaman Y (2015). Social media adoption: the role of media needs and innovation characteristics. Comput Human Behav.

[66] Brouwer W, Kroeze W, Crutzen R, de Nooijer J, de Vries NK, Brug J, Oenema A (2011). Which intervention characteristics are related to more exposure to internet-delivered healthy lifestyle promotion interventions? A systematic review. J Med Internet Res.

[67] Brouwer W, Oenema A, Crutzen R, de Nooijer J, de Vries NK, Brug J (2008). An exploration of factors related to dissemination of and exposure to internet-delivered behavior change interventions aimed at adults: a Delphi study approach. J Med Internet Res.

[68] Brouwer W, Oenema A, Crutzen R, de Nooijer J, de Vries N, Brug J (2009). What makes people decide to visit and use an internet‐delivered behavior‐change intervention?. Health Education.

[69] Dawot N, Ibrahim R (2014). A review of features and functional building blocks of social media. Proceedings of the 2014 8th Malaysian Software Engineering Conference (MySEC).

[70] Elaheebocus SM, Weal M, Morrison L, Yardley L (2018). Peer-based social media features in behavior change interventions: systematic review. J Med Internet Res.

[71] Ko N, Hsieh C, Wang M, Lee C, Chen C, Chung A, Hsu S (2013). Effects of internet popular opinion leaders (iPOL) among Internet-using men who have sex with men. J Med Internet Res.

[72] Maloney S, Tunnecliff J, Morgan P, Gaida JE, Clearihan L, Sadasivan S, Davies D, Ganesh S, Mohanty P, Weiner J, Reynolds J, Ilic D (2015). Translating evidence into practice via social media: a mixed-methods study. J Med Internet Res.

[73] Michie S, Richardson M, Johnston M, Abraham C, Francis J, Hardeman W, Eccles MP, Cane J, Wood CE (2013). The behavior change technique taxonomy (v1) of 93 hierarchically clustered techniques: building an international consensus for the reporting of behavior change interventions. Ann Behav Med.

[74] Simeon R, Dewidar O, Trawin J, Duench S, Manson H, Pardo Pardo J, Petkovic J, Hatcher Roberts J, Tugwell P, Yoganathan M, Presseau J, Welch V (2020). Behavior change techniques included in reports of social media interventions for promoting health behaviors in adults: content analysis within a systematic review. J Med Internet Res.

[75] Tajudeen FP, Jaafar NI, Ainin S (2018). Understanding the impact of social media usage among organizations. Inf Manag.

[76] Tunnecliff J, Weiner J, Gaida JE, Keating JL, Morgan P, Ilic D, Clearihan L, Davies D, Sadasivan S, Mohanty P, Ganesh S, Reynolds J, Maloney S (2017). Translating evidence to practice in the health professions: a randomized trial of Twitter vs Facebook. J Am Med Inform Assoc.

[77] Wirtz B, Piehler R, Ullrich S (2013). Determinants of social media website attractiveness. J Electronic Commerce Res.

[78] Barton C (2017). The current sports medicine journal model is outdated and ineffective. Aspetar Sports Med J.

[79] Gates A, Featherstone R, Shave K, Scott SD, Hartling L (2018). Dissemination of evidence in paediatric emergency medicine: a quantitative descriptive evaluation of a 16-week social media promotion. BMJ Open.

[80] Sbaffi L, Rowley J (2017). Trust and credibility in web-based health information: a review and agenda for future research. J Med Internet Res.

[81] (2016). Guidance for the Assessment of Context and Implementation in Health Technology Assessments (HTA) and Systematic Reviews of Complex Interventions: The Context and Implementation of Complex Interventions (CICI) Framework.

[82] Chapman SJ, Grossman RC, FitzPatrick ME, Brady RR (2019). Randomized controlled trial of plain English and visual abstracts for disseminating surgical research via social media. Br J Surg.

[83] Ibrahim AM, Lillemoe KD, Klingensmith ME, Dimick JB (2017). Visual abstracts to disseminate research on social media: a prospective, case-control crossover study. Ann Surg.

[84] Fudan University Evidence-Based Nursing Center Anual Report. Yunzhan.

[85] It doesn't have to hurt team. Dr. Christine Chambers.

[86] Levac D, Glegg SM, Camden C, Rivard LM, Missiuna C (2015). Best practice recommendations for the development, implementation, and evaluation of online knowledge translation resources in rehabilitation. Phys Ther.

[87] Kitson A, Brook A, Harvey G, Jordan Z, Marshall R, O'Shea R, Wilson D (2018). Using complexity and network concepts to inform healthcare knowledge translation. Int J Health Policy Manag.

[88] Greenhalgh T, Wherton J, Papoutsi C, Lynch J, Hughes G, A'Court C, Hinder S, Fahy N, Procter R, Shaw S (2017). Beyond adoption: a new framework for theorizing and evaluating nonadoption, abandonment, and challenges to the scale-up, spread, and sustainability of health and care technologies. J Med Internet Res.

